# Eat Healthy to Live Healthy: Habits and Trends

**DOI:** 10.3390/ijerph17249422

**Published:** 2020-12-16

**Authors:** Antonio Di Mauro, Maria Elisabetta Baldassarre, Nicola Laforgia

**Affiliations:** Department of Biomedical Science and Human Oncology, “Aldo Moro” University of Bari, 70124 Bari, Italy; mariaelisabetta.baldassarre@uniba.it (M.E.B.); nicola.laforgia@uniba.it (N.L.)

“Eat healthy to live healthy” is a fundamental mantra for long-term wellbeing. This Special Issue was developed to address challenges in prevention strategies based on nutritional interventions and lifestyle changes throughout one’s life, from fetus to adulthood. In fact, even maternal nutrition, a well-known risk factor for pregnancy complications, has become an apparent determinant of long-term inter-generational health problems in offspring [1].

The provision of appropriate nutrition is crucial during infancy and childhood, a period of life characterized by rapid growth and development but also long-term functional outcomes. In this context, the duration of breastfeeding and complementary feeding exerts an important role in establishing flavor preferences and behaviors, which may, in turn, reduce the risk of chronic illnesses such as overweight or obesity.

During the first months of life, breastfeeding is the most important factor in preventing obesity in childhood because of the low protein content and epigenetic effects of breast milk.

It is very important to help mothers in initiating and continuing breastfeeding by providing them with accurate information during pregnancy and after delivery. Stressful events can negatively affect the mother when she begins breastfeeding. A recent study by Foligno et al. highlights that the stress caused by hospitalization can influence the success of breastfeeding, particularly in intensive care settings and during long hospital stays [2].

After the first year of life, the baby’s nutrition begins to increasingly resemble that of other family members. Attention must still be paid to choosing foods that meet the specific safety and quality requirements of early childhood. Unfortunately, the food consumption behaviors of families have often adapted to routines, which can have adverse effects on health. Calorie-rich foods with poor nutritional value are often more appealing and preferred to healthy foods that are rich in micronutrients; consequently, there has been a rise in non-communicable chronic diseases, especially in developing countries.

In the review by D’Auria et al., the presented evidence addresses the effects of complementary feeding on the onset of chronic non-communicable diseases, and practical tips for a healthy approach to complementary feeding are discussed [3].

Furthermore, diets that are perceived to be healthier are being increasingly adopted during complementary feeding without the supervision of expert physicians, as illustrated in a population-based survey by Baldassarre et al. In such cases, potential detrimental long-term effects due to severe nutritional deficiencies need to be considered [4].

Alcohol consumption during adolescence is prevalent among unhealthy lifestyles that are adopted throughout life. Excessive drinking is a major cause of health problems worldwide, including “binge drinking”, the consumption of an excessive amount of alcohol in a short period of time. A study by Kim et al. demonstrated that 52% of Korean university students, both male and female, reported binge drinking, and this may result in acute and chronic health problems, involvement in various accidents, and anti-social behaviors [5]. The authors also found a significant relationship between physical activity and alcohol consumption and suggested alcohol-abuse prevention strategies based on other observations.

The consumption of health foods can reduce costs and improve overall health and quality of life. In adulthood, workplaces with facilities that serve food to employees (staff canteens) for meals during their working hours can offer healthy, well-balanced food according to modern guidelines and increase the development of good eating habits.

A recent paper by Czarniecka-Skubina et al. [6] underlined the importance of workplace nutrition as a potential key intervention to reduce the incidence of chronic diseases in adult populations and improve public health via direct and indirect measures. The authors noted the importance of taking breaks, which plays a role in emotional exhaustion, job satisfaction, and organizational behavior. The authors conducted their research in five staff canteens in Warsaw that were similar in size, the number of seats for consumers, and the number of meals served daily. Their results have practical implications for owners or managers of staff canteens: customers follow trends regarding “healthy” diets and expect canteens to shift toward offering healthy meals, emphasizing the need to increase the variety of meals, including the availability of vegetarian, vegan, and health-promoting dishes.

Moreover, health food marketing strategies and interventions for high-risk populations need to be improved to promote public health behaviors.

In this context, the results of an interesting study by Lee et al. [7] can be used as a reference for health food marketing strategies. The authors targeted people who had purchased health food products to improve their gastrointestinal functions. The authors demonstrated that perceptions of susceptibility, severity, benefits of action, and behavioral control exerted a positive influence on repurchase intention.

For the development of health food marketing strategies, the rapidly expanding market for dietary supplements (soft capsules, hard capsules, tablets, granules, or liquids that contain functional physiological ingredients differing from those in regular food) is of notable interest, as demonstrated in the study by Sato et al. [8].

A food supplement, considered a form of self-medication with the potential to reduce the public health burden, is expected to contribute to promoting health and preventing disease in aging societies. Dietary supplements are positioned between food and medicine in the regulatory spectrum. Good manufacturing practice (GMP) is advocated and implemented as a standardized procedure for manufacturing dietary supplements. The Japanese study by Sato et al. reveals that pharmaceutical capability influences the quality level in the manufacturing of dietary supplements, accompanied by expertise in manufacturing a wide range of dietary supplement products.

Natural and dietary alternatives to the management of non-communicable chronic diseases have to be further explored. The consumption of health foods and the use of natural alternatives to drugs, such as nutraceuticals and functional foods, can reduce costs and improve overall health and quality of life [9]. In the review by Idrus et al., the available evidence showed that honey had a protective effect on cardiovascular disease, a major public health burden worldwide [10].

In a paper by Baldassarre et al., alginate-based formulations from brown algae were found to be effective as a first treatment option for persistent gastrointestinal distress related to gastroesophageal reflux (GER) in infants and may reduce the use of other treatments and tests [11].

Nutritional interventions and lifestyle have established their potential roles in the prevention of human disease at all stages of life. This Special Issue discusses different habits and trends to help physicians in their daily practices and assist governments in the promotion of preventive health measures.

## References

[1] Ciccone M.M., Scicchitano P., Salerno C., Gesualdo M., Fornarelli F., Zito A., Filippucci L., Riccardi R., Cortese F., Pini F. (2013). Aorta structural alterations in term neonates: The role of birth and maternal characteristics. Biomed. Res. Int..

[2] Foligno S., Finocchi A., Brindisi G., Pace A., Amadio P., Dall’Oglio I., Portanova A., Rossi P., Dotta A., Salvatori G. (2020). Evaluation of Mother’s Stress during Hospitalization Can Influence the Breastfeeding Rate. Experience in Intensive and Non Intensive Departments. Int. J. Environ. Res. Public Health.

[3] D’Auria E., Borsani B., Pendezza E., Bosetti A., Paradiso L., Zuccotti G.V., Verduci E. (2020). Complementary Feeding: Pitfalls for Health Outcomes. Int. J. Environ. Res. Public Health.

[4] Baldassarre M.E., Panza R., Farella I., Posa D., Capozza M., Di Mauro A., Laforgia N. (2020). Vegetarian and Vegan Weaning of the Infant: How Common and How Evidence-Based? A Population-Based Survey and Narrative Review. Int. J. Environ. Res. Public Health.

[5] Kim Y., Kuan G. (2020). Relationship between Alcohol Consumption and Drinking Refusal Self-Efficacy among University Students: The Roles of Sports Type and Gender. Int. J. Environ. Res. Public Health.

[6] Czarniecka-Skubina E., Górska-Warsewicz H., Trafiałek J. (2020). Attitudes and Consumer Behavior toward Foods Offered in Staff Canteens. Int. J. Environ. Res. Public Health.

[7] Lee K.-Y., Wei C.-Y., Wu M.-H., Hsieh C.-M. (2020). Determinants of the Public Health Promotion Behavior: Evidence from Repurchasing Health Foods for Improving Gastrointestinal Tract Functions. Int. J. Environ. Res. Public Health.

[8] Sato K., Kodama K., Sengoku S. (2020). Corporate Characteristics and Adoption of Good Manufacturing Practice for Dietary Supplements in Japan. Int. J. Environ. Res. Public Health.

[9] Indrio F., Di Mauro A., Riezzo G., Panza R., Cavallo L., Francavilla R. (2015). Prevention of functional gastrointestinal disorders in neonates: Clinical and socioeconomic impact. Benef. Microbes.

[10] Bt Hj Idrus R., Sainik N.Q.A.V., Nordin A., Saim A.B., Sulaiman N. (2020). Cardioprotective Effects of Honey and Its Constituent: An Evidence-Based Review of Laboratory Studies and Clinical Trials. Int. J. Environ. Res. Public Health.

[11] Baldassarre M.E., Di Mauro A., Pignatelli M.C., Fanelli M., Salvatore S., Di Nardo G., Chiaro A., Pensabene L., Laforgia N. (2020). Magnesium Alginate in Gastro-Esophageal Reflux: A Randomized Multicenter Cross-Over Study in Infants. Int. J. Environ. Res. Public Health.

